# ICS II protects against cardiac hypertrophy by regulating metabolic remodelling, not by inhibiting autophagy

**DOI:** 10.1111/jcmm.16175

**Published:** 2020-12-08

**Authors:** Dongjian Han, Bo Wang, Xinyue Cui, Weiwei He, Yi zhang, Qingjiao Jiang, Fuhang Wang, Zhiyu Liu, Deliang Shen

**Affiliations:** ^1^ Department of Cardiology The First Affiliated Hospital of Zhengzhou University Zhengzhou China

**Keywords:** autophagy, cardiac hypertrophy, Icariside II, ketone body, SIRT3

## Abstract

Cardiac hypertrophy is characterized by a shift in metabolic substrate utilization. Therefore, the regulation of ketone body uptake and metabolism may have beneficial effects on heart injuries that induce cardiac remodelling. In this study, we investigated whether icariside II (ICS II) protects against cardiac hypertrophy in mice and cardiomyocytes. To create cardiac hypertrophy animal and cell models, mice were subjected to transverse aortic constriction (TAC), and embryonic rat cardiomyocytes (H9C2) were stimulated with angiotensin II, a neurohumoral stressor. Both the in vivo and in vitro results suggest that ICS II treatment ameliorated pressure overload–induced cardiac hypertrophy and preserved heart function. In addition, apoptosis and oxidative stress were reduced in the presence of ICS II. Moreover, ICS II inhibited excess autophagy in TAC‐induced hearts and angiotensin II–stimulated cardiomyocytes. Mechanistically, we found that ICS II administration regulated SIRT3 expression in cardiac remodelling. SIRT3 activation increased ketone body transportation and utilization. Collectively, our data show that ICS II attenuated cardiac hypertrophy by modulating ketone body and fatty acid metabolism, and that this was likely due to the activation of the SIRT3‐AMPK pathway. ICS II treatment may provide a new therapeutic strategy for improving myocardial metabolism in cardiac hypertrophy and heart failure.

## 1 INTRODUCTION

Heart failure (HF) is one of the most common causes of hospital admission and readmission in patients over 65 years of age. It is a clinical syndrome resulting from structural and functional cardiac disorders that impair the heart's ability to fill with (diastolic) and/or to eject (systolic) blood commensurate with the metabolic needs of the body.^1^, ^2^, ^3^ A common precursor and contributor to HF is cardiac hypertrophy, a pathological condition that sets in as the heart attempts to compensate for its poor ability to circulate the blood. Cardiac hypertrophy is characterized by an increase in the size of individual cardiomyocytes, rather than their overall numbers, in order to temporarily maintain the cardiac output.^4^ Long‐term hypertrophy eventually leads to ischaemia, arrhythmia, heart failure and sudden death.^5^ However, the precise pathophysiological mechanisms that explain the transition from hypertrophy to clinical HF are not well understood.^6^

Autophagy plays an important role in cellular homeostasis and metabolism balance under basal conditions.^7^, ^8^ It also serves as a protective mechanism against cellular stress by eliminating misfolded proteins and damaged organelles to provide raw materials and energy for the synthesis of critical cellular factors.^9^ The regulation of autophagy has been suggested as a potential target for the treatment of cardiac dysfunction.^10^, ^11^

Pathological cardiac hypertrophy, as an adaptive and compensatory remodelling of the heart, occurs in response to sustained blood pressure overload,^12^ a condition that occurs in pathologies such as long‐standing systemic arterial hypertension or LV out‐flow tract obstruction. During this remodelling process, the energy demands of the myocardium increase.^13^, ^14^ The heart uses a variety of substrates to generate energy, including exogenous fatty acids, glucose, lactate and amino acid.^15^ Although the roles of these primary myocardial fuels are well established, the influence of myocardial ketone bodies still remains poorly understood. Recent evidence has shown that patients with heart failure experience an increase in the uptake of ketone bodies. These observations suggest that the hypertrophied or failing hearts shift to ketone bodies as a significant fuel source for ATP production.^16^, ^17^ A better understanding of how adaptive or maladaptive hearts utilize ketone bodies may open up new therapeutic avenues to treat cardiac hypertrophy.

SIRT3, a highly conserved nicotinamide adenine dinucleotide (NAD^+^)–dependent histone deacetylase, primarily expressed in mitochondria, deacetylates and activates global mitochondrial proteins leading to metabolic activation.^18^ It induces ketone body production by increasing the expression of the 3‐hydroxy‐3‐methylglutaryl (HMG)‐CoA synthase 2 (HMGCS2) gene during fasting.^19^ AMP‐activated protein kinase (AMPK) also participates in many metabolic processes and regulates the production and utilization of ketone bodies in response to cardiac hypertrophy.^20^ Activating the SIRT3‐AMPK pathway could provide the heart with more fuel and more energy to help it adapt to sustained stress.

Icariside II (ICS II) is one of the main active flavonoid glycosides derived from *Herba Epimedii,* a traditional Chinese herbal medicine used for the treatment of a range of clinical diseases, including cerebral ischaemia‐reperfusion injury, erectile dysfunction and dementia.^21^, ^22^ In addition, though less numerous, there are scientific studies indicating that ICS II ameliorates pressure overload–induced cardiac remodelling.^23^ However, the precise mechanisms of action have not been elucidated, and the ability of ICS II to regulate autophagy and myocardial metabolism has not been well documented.

In this study, we investigated whether ICS II can protect against transverse aortic constriction (TAC)–induced and angiotensin II (Ang II)–induced cardiac remodelling and hypertrophy by regulating autophagy and ketone body metabolism through the SIRT3‐AMPK pathway.

## 2 METHODS

### 2.1 Antibodies and reagents

ICS II (purity ≥ 98%), rapamycin (Rapa) and chloroquine (CQ) were purchased from MedChemExpress CO. (Monmouth Junction) and solubilized in 0.1% dimethyl sulphoxide (DMSO; Sigma, USA). Angiotensin II (Ang II) was purchased from Sigma‐Aldrich (St. Louis). Rabbit anti‐Nox2/gp91phox monoclonal antibody (Abcam, ab129068), rabbit anti‐NADPH oxidase 4 antibody (Abcam, ab133303), mouse anti‐haeme oxygenase 1 antibody (Abcam, ab13248), rabbit anti‐Bcl‐2 antibody (Abcam, ab32124) and rabbit anti‐Bax antibody (Abcam, ab32503) were purchased from Abcam. Rabbit anti‐LC3 I/II antibody (#4108), rabbit anti‐Atg5 antibody (#9980), rabbit anti‐Atg7 antibody (#8558), rabbit anti‐Beclin 1 antibody (#3495), rabbit anti‐p62 antibody (#23214), rabbit anti‐ACC antibody (#3662), rabbit anti‐SIRT3 antibody (#2627), rabbit anti‐p‐ACC antibody (#11818), rabbit anti‐AMPK antibody (#2532), rabbit anti‐p‐AMPK antibody (#50081), rabbit anti‐cleaved caspase 3 antibody (#9664) and rabbit anti‐GAPDH antibody were purchased from CST. Rabbit anti‐OXCT1 antibody (#12175‐1‐AP) and rabbit anti‐MCT1 antibody (#20139‐1‐AP) were purchased from Proteintech.

### 2.2 Animal protocols

All experimental procedures involving animals in this study were performed according to the Guide for the Care and Use of Laboratory Animals published by the US National Institutes of Health (National Institutes of Health Publication 85‐23, revised 1996) and were approved by the Experimental Animals Ethics Committee of Zhengzhou University (Zhengzhou, China). Male C57BL/6 mice (20‐22 g) were obtained from the Animal Research Center of Henan Province, housed in an animal care facility with 12‐h light‐dark cycles and allowed free access to a rodent diet and tap water. Transverse aortic constrictions (TAC) were performed as previously described.^24^ In brief, mice were anaesthetized by intraperitoneal injection of a mixture of 100 mg/kg ketamine and 5 mg/kg xylazine, orally intubated with 20‐gauge tubing and placed on a MiniVent Type 845 respirator (Harvard, USA) operating at 100‐120 breaths per minute (0.15 ml tidal volume). The chest was opened at the second intercostal space in a sterile manner. The aortic arch was ligated between the innominate and left common carotid arteries with 7‐0 silk sutures. Before ligating, a 27‐gauge needle was inserted within the knot and pressed up against the aortic arch. The knot was then tied, and the needle was subsequently removed. After the procedure, the chest wall was sutured closed and each mouse was monitored for 24 h on a heating pad. Mice in the sham group were subjected to the same operation but did not have their aortic arches ligated. The mortality rate was about 5% after 8 weeks. Icariside II (purity > 98%) was administered to the mice orally, using a gavage needle, at a dose of 16 mg/kg/d, 3 times per week, for 8 weeks. Mice in the sham group, and in the group that just receive TAC surgery, received saline in volumes equal to that of mice in the treatment groups. Chloroquine (10 mg/kg) and rapamycin (10 mg/kg) were injected intraperitoneally once a day for 8 weeks.

### 2.3 Echocardiography

Once anaesthetized, the mice were put on a temperature‐controlled imaging table in the supine position. Transthoracic ultrasonographies were performed with the Vevo 2100 micro‐ultrasound (VisualSonics) to measure the cardiac function and the thickness of the ventricular walls. The left ventricular ejection fraction (LVEF), left ventricular fractional shortening (LVFS), left ventricular anterior wall thickness at end‐diastole (LVAWd), left ventricular anterior wall thickness at end‐systole (LVAWs), left ventricular posterior wall thickness at end‐diastole (LVPWd) and left ventricular posterior wall thickness at end‐systole (LVPWs) were measured and analysed by an echocardiographer who was blind to the experiment protocols and heart condition.

### 2.4 Histochemistry and immunohistochemistry

The entire heart of each anaesthetized mouse was harvested and fixed in 4% paraformaldehyde, dehydrated and then embedded in paraffin. Heart tissues were cut into sections (5 μm) and stained using Masson's trichrome or tagged with immunofluorescent antibodies. Representative images were captured and analysed.

### 2.5 Cell culture and treatment

The H9C2 cell line was purchased from ATCC (CRL‐1446). Cells were cultured in Dulbecco's modified Eagle's medium (DMEM) supplemented with 10% foetal bovine serum (FBS), 100 U/ml penicillin and 100 g/ml streptomycin in 25‐cm^2^ tissue culture flasks at 37°C in a humidified atmosphere containing 5% CO_2_. Rapa was added at the concentration of 0.5 mmol/L for 2 h before Ang II (20 μmol/L) and incubated for another 24 h.

### 2.6 Oxidative stress detection

The total superoxide dismutase (SOD) and malondialdehyde (MDA) in the myocardium were analysed using commercial assay kits (Beyotime) according to the manufacturer's instructions. ROS production was detected by DCFH‐DA staining in vitro. Briefly, cells were incubated with DCFH‐DA (5 μmol/L) at 37°C for 30 minutes. Fluorescent images were observed using a laser scanning confocal microscope (Zeiss LSM 880) and analysed using ImageJ software.

### 2.7 Small interfering RNA

H9C2 cells were transiently transfected with SIRT3 small interfering RNA (siRNA) or negative control (NC) siRNA (GenePharma) using the Lipofectamine 3000 Transfection Reagent (Invitrogen) according to the manufacturer's instructions.

### 2.8 Immunofluorescent staining

Immunofluorescent staining was performed according to our previous description.^25^, ^26^ In briefly, H9C2 cells were washed three times using phosphate‐buffered saline (PBS), fixed in 4% paraformaldehyde for 30 minutes, permeabilized with 0.1% Triton X‐100 for 10 minutes and blocked for 1 hour with goat serum. Next, the cells were incubated with anti‐LC3 II (1:500), anti‐OXCT1 (1:100), and anti‐MCT1 (1:100) antibodies overnight at 4°C. Then, the cells were incubated with goat anti‐rabbit IgG (H + L) secondary antibodies (Alexa Fluor 488 or Alexa Fluor 594) for 1 hour. Cell nuclei were counterstained with 4’,6‐diamidino‐2‐phenylindole (DAPI) (Beyotime Inc, Haimen, China). The cells were observed using a laser scanning confocal microscope (Zeiss LSM 880), and the resulting fluorescent images were analysed using ImageJ.

### 2.9 Molecular docking protocol

Molecular docking between ICS II and SIRT3 was performed using AutoDock Vina 1.1.2 and AutoDockTools 1.5.6. The X‐ray crystal structure of SIRT3 (PDB ID: 4C7B) was obtained from the Protein Data Bank (PDB) archives of the Research Collaboratory for Structural Bioinformatics (RCSB) and used as the target for molecular docking. The 2D structure of ICS II was downloaded from PubChem (RRID:SCR_004284) and then converted into a 3D structure. The protein‐ligand complex interactions were studied using the AutoDock Vina 1.1.2 system.

### 2.10 Western blots

Soon after extracting the mouse hearts, myocardial proteins were isolated, quantified by bicinchoninic acid (BCA) assay, separated by SDS‐PAGE and transferred to polyvinylidene difluoride membranes. The membranes were blocked with 5% non‐fat milk at room temperature and incubated with primary antibodies overnight at 4°C. When washed 3 times with TBST (150 mmol/L NaCl, 50 mmol/L Tris [PH 7.5] and 0.1% Tween‐20), the corresponding horseradish peroxidase (HRP)–conjugated secondary antibodies were incubated with the membranes for 1 hour at 37°C. Immunoreactive bands were visualized with enhanced chemiluminescent (ECL) reagent and analysed using ImageJ software. GAPDH or β‐actin was used as the internal loading control.

### 2.11 qRT‐PCR analysis

Total RNA was extracted from heart tissue with TRIzol Reagent (Invitrogen, Carlsbad, CA, USA) according to the manufacturer's instructions. Then, the cDNA was reverse transcribed with the PrimeScript^TM^ RT Reagent Kit (TaKaRa). The relative expression of genes was evaluated with real‐time PCR using the ABI Prism 7500 Sequence Detection System (Applied Biosystems). The expression levels of target genes were normalized to GAPDH gene expression.

### 2.12 Statistical analysis

All data are presented as mean ± SEM Comparisons between more than two groups were conducted using one‐way or two‐way ANOVA with Bonferroni's post hoc testing. For comparisons between two groups, a standard Student's *t* test was used. *P* < .05 was considered statistically significant. All statistical analyses were performed with GraphPad Prism 8.0 software.

## 3 RESULTS

### 3.1 ICS II improves cardiac function and attenuates cardiac remodelling in TAC‐induced hypertrophy

**Figure 1:**
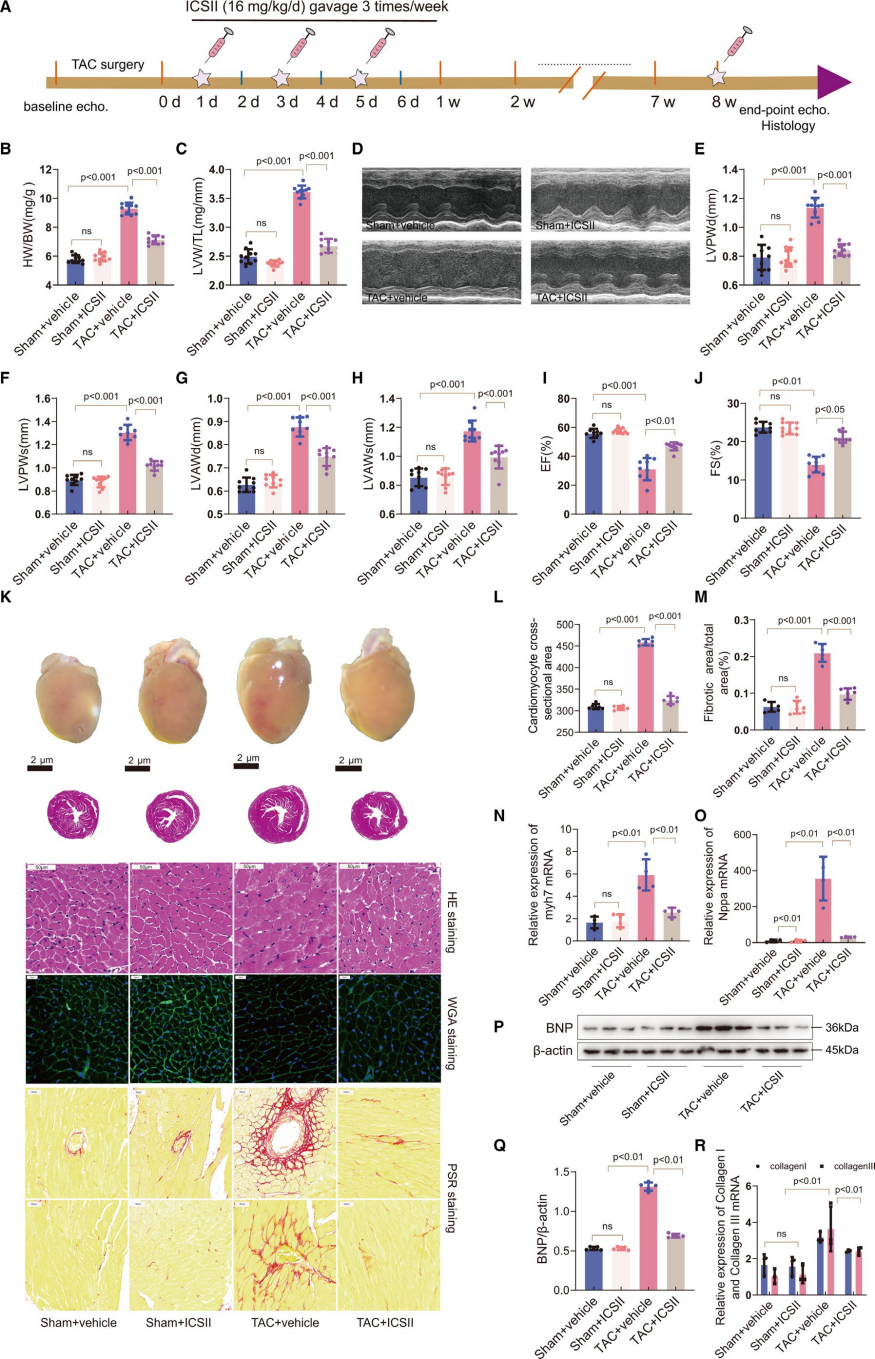
ICS II attenuated pressure overload–induced cardiac hypertrophy. A, Timeline of study protocol. B, C, The HW/BW and LVW/TL ratios for the sham and TAC‐induced mice after ICS II treatment or vehicle administration. n = 10/group. D–J, 8 weeks after TAC surgery, the following mouse cardiac functions were assessed: the left ventricle posterior wall thickness in diastole (LVPWd), left ventricle posterior wall thickness in systole (LVPWs), left ventricle anterior wall thickness in diastole (LVAWd), left ventricle anterior wall thickness in systole (LVAWs), ejection fraction (EF%) and fractional shortening (FS%). n = 10/group. K, Representative photographs of the morphology of the hearts from each group (first row), haematoxylin and eosin (H&E)–stained micrographs (second and third rows: scale bar = 2μm and 50μm), wheat germ agglutinin (WGA)–stained micrographs (fourth row: scale bar = 20μm) and picrosirius red (PSR)–stained micrographs showing perivascular and myocardial fibrosis (fifth row and sixth row: scale bar = 50μm). n = 6/group. L, Quantification of the cardiomyocyte cross‐sectional areas. n = 6/group. M, Quantification of myocardial fibrosis. n = 6/group. N, O, Real‐time PCR results for myocardial Myh7 and Nppa expression. n = 4/group. P, Western blot of myocardial BNP in mice with or without ICS II treatment. Q, Quantification of BNP expression. R, Real‐time PCR results for myocardial procollagen I/III expression. n = 4/group

To characterize the role of ICS II in TAC‐induced cardiac hypertrophy, C57BL/6 mice were treated with ICS II at a dose of 16 mg/kg/d, three times per week, starting on the day after surgery (Figure 1A). After 8 weeks of TAC‐induced pressure overload, mice treated with ICS II exhibited smaller increases in heart weight (HW)/bodyweight (BW) ratio, as well as left ventricular weight (LVW)/tibia length (TL) ratio (Figure 1B‐C), compared with those that did not receive ICS II treatment.

Echocardiography showed an increase in the diastolic and systolic LV posterior and anterior wall thicknesses (left ventricle posterior wall thickness in diastole (LVPWd), left ventricle posterior wall thickness in systole (LVPWs), left ventricle anterior wall thickness in diastole (LVAWd), left ventricle anterior wall thickness in systole (LVAWs)) in TAC‐induced hypertrophic mice. However, ICS II treatment significantly suppressed the increase in LV wall thickness (Figure 1D‐H). Moreover, in mice treated with ICS II, the values for left ventricular ejection fraction (LVEF) and left ventricular fractional shortening (LVFS) were significantly higher than in mice that underwent TAC but did not receive ICS II treatment (Figure 1I‐J).

Compared to the sham group, mice that underwent TAC developed larger heart sizes. This growth in heart size was significantly attenuated by ICS II treatment (Figure 1K). Histological examination after haematoxylin and eosin (H&E) and wheat germ agglutinin (WGA) staining also showed that TAC‐induced cardiac hypertrophy was markedly ameliorated by ICS II treatment (Figure 1K‐L). The hearts’ relative expression of genes related to cardiac hypertrophy was analysed. As shown in Figure 1N‐O, the TAC‐induced increase in Nppa and Myh7 mRNA expression in the myocardium was significantly attenuated in mice treated with ICS II. In addition, picrosirius red (PSR) staining revealed that the areas of interstitial and perivascular fibrosis were significantly less prominent in TAC‐induced hypertrophic mice treated with ICS II compared with those in the vehicle‐treated group (Figure 1K‐M). Furthermore, Western blot analysis showed that brain natriuretic peptide (BNP), a hypertrophic marker, was down‐regulated in mice treated with ICS II, compared with those in the TAC + vehicle group (Figure 1P‐Q). Real‐time PCR demonstrated that ICS II also markedly down‐regulated the expression of procollagen I/III genes after TAC (Figure 1R). In conclusion, these results indicate that ICS II is a potential cardioprotective agent that can attenuate TAC‐induced cardiac hypertrophy.

### 3.2 ICS II ameliorated TAC‐induced oxidative stress and myocardial apoptosis

**Figure 2:**
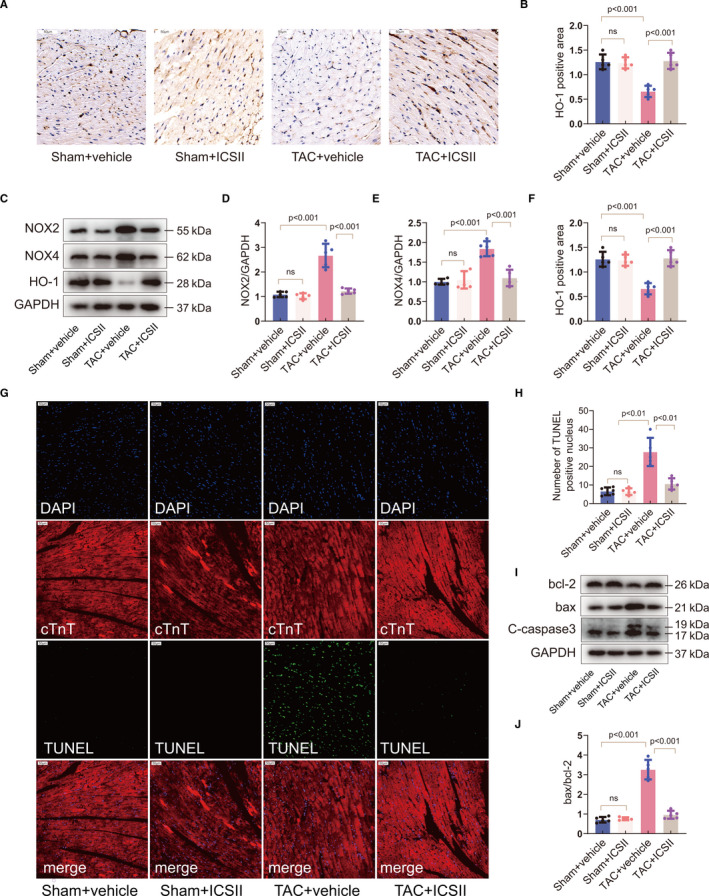
ICS II inhibits oxidative stress and cardiac apoptosis. A, B, Immunohistochemical analyses of HO‐1 expression in the myocardium. Scale bar = 50μm. n = 6/group. C–F, Western blots and quantification of NOX2, NOX4 and HO‐1 expression. n = 5. G, H, Representative micrographs and quantification of TUNEL‐positive cardiomyocytes in the mouse myocardium. Scale bar = 50μm. n = 6. I, J, Western blots of cleaved caspase 3 and bax/bcl‐2 and quantification of bax/bcl‐2 expression. n = 5

The production of reactive oxygen species (ROS) plays a critical role in TAC‐induced cardiac hypertrophy. Previous research has reported that the up‐regulation of haeme oxygenase‐1 (HO‐1) may be cardioprotective by inhibiting oxidative stress.^27^ In order to test the ability of ICS II to suppress oxidative stress induced by TAC surgery, the myocardial expression of HO‐1 was measured via Western blot and immunohistochemistry. The results indicate that HO‐1 was up‐regulated in the hearts of mice that received ICS II, compared with those that did not (Figure 2A‐C and F). Next, we sought to test whether ICS II could inhibit the expression of nicotinamide adenine dinucleotide phosphate (NADPH) oxidase (NOX). The NADPH oxidase complex with NOX2 and NOX4 is considered to be the main source of ROS production through oxidizing intracellular NADPH/NADH, causing electron transportation across the membrane and reduction of molecular oxygen into superoxide.^28^ As shown in Figure 2C, the expression of NADPH oxidase 2 and NADPH oxidase 4 (NOX2; NOX4) increased after TAC, and significantly decreased after ICS II treatment (Figure 2C‐E). Moreover, quantitative analysis showed enhanced myocardial superoxide dismutase (SOD) levels and reduced malondialdehyde (MDA) levels after ICS II treatment (Figure S1A‐B). All of these results demonstrate the anti‐oxidative effects of ICS II on TAC‐induced cardiac hypertrophy.

In addition to ROS, apoptosis plays an important role in the pathology and physiology of heart diseases. The bax/bcl‐2 axis is considered as a main pathway in regulating intracellular oxidative stress and cell death. Bcl‐2 could protect the cell against apoptotic stimuli, whereas bax activation accelerates cell death.^29^ In the present study, the increase in cellular apoptosis observed in the mouse myocardium after TAC surgery was reduced after ICS II treatment (Figure 2G‐H). Additionally, ICS II remarkably decreased the bax/bcl‐2 ratio and the expression of cleaved caspase 3 (Figure 2I‐J and Figure S1C). Taken as a whole, these findings indicate that ICS II can reduce myocardial apoptosis by regulating the bcl‐2 family of proteins.

### 3.3 ICS II inhibits hypertrophy in Ang II–stimulated cardiomyocyte cultures

**Figure 3:**
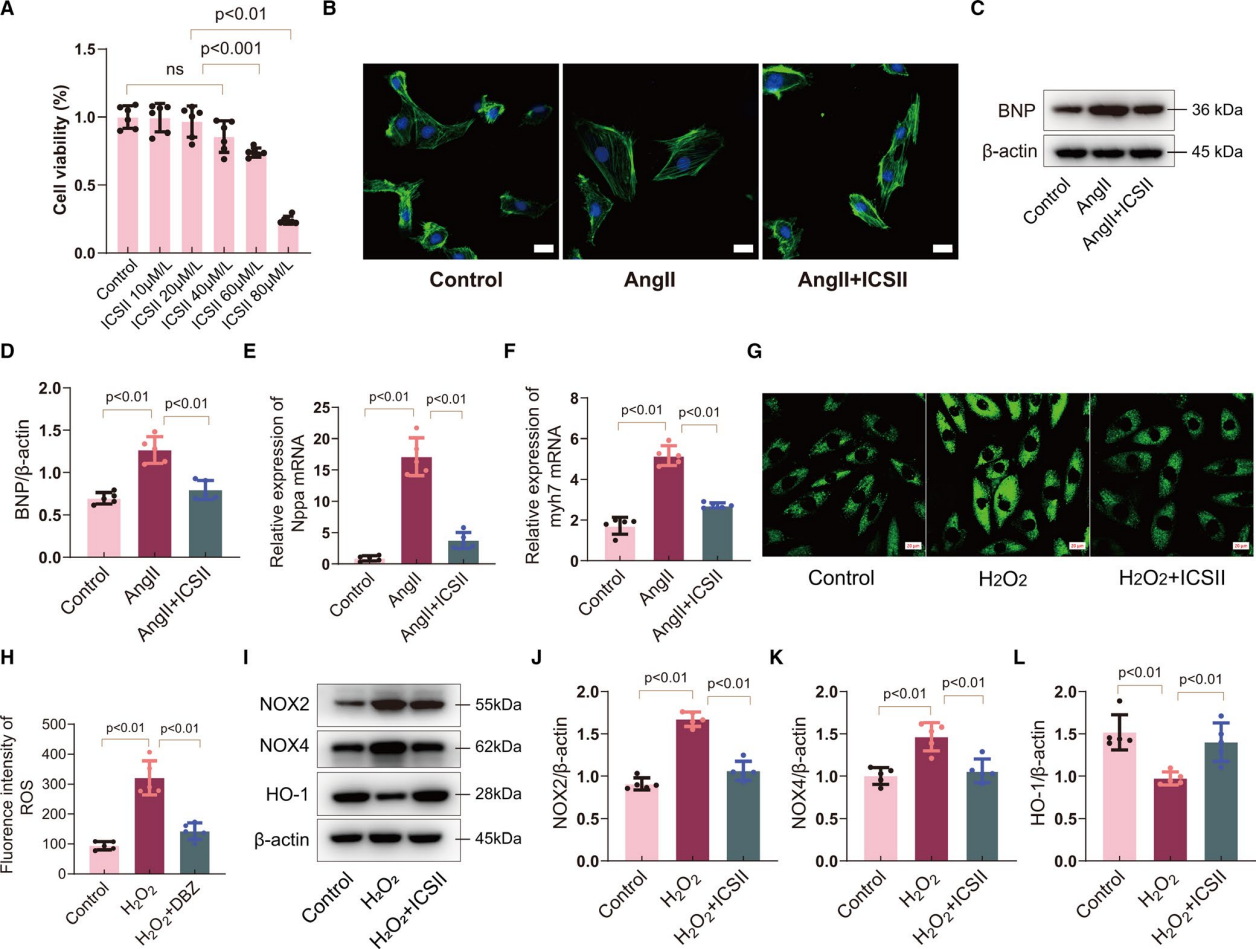
ICS II inhibited cardiomyocyte hypertrophy and oxidative stress in vitro. A, Quantification of the viability of cardiomyocytes treated with different concentrations of ICS II. n = 6. B, Representative micrographs of cardiomyocytes as a result of ICS II (20 μmol/L) and Ang II incubation for 24 h. n = 10. Scale bar = 20μm. C, D, Western blot for BNP expression in cardiomyocytes and quantification. n = 5. E, F, Real‐time PCR analyses of the expression of hypertrophic markers Nppa and Myh7. n = 5. G, H, DCFH‐DA staining detection of ROS production and quantification. n = 5. H–K, Western blots for Nox2, Nox4 and HO‐1, and quantification. n = 5

The effects of ICS II on cardiomyocyte survival were investigated in vitro by subjecting the cells to increasing concentrations of the therapeutic. After 24 h, ICS II exerted no effect on cardiomyocytes at lower concentrations (10‐40 μM/L), but dramatically decreased cell survival at higher concentrations (60‐80 μM/L) (Figure 3A). The cardiomyocytes were then exposed to Ang II, a known hypertrophic inducer. Immunostaining with phalloidin revealed that treating the Ang II–induced hypertrophic cells with ICS II reduced their size (Figure 3B and Figure S2A). Consistent with the in vivo results, the expression of the cardiac hypertrophy–related molecular marker BNP was higher after Ang II stimulation. However, the increase in expression was prevented by ICS II treatment (Figure 3C‐D). In addition, after 24 h, the levels of Nppa and Myh7 were dramatically lower in cells treated with ICS II (20 μmol/L) than in those cultured with Ang II alone (Figure 3E‐F). These data indicate that ICS I inhibited Ang II–induced cardiomyocyte hypertrophy.

### 3.4 ICS II attenuated H_2_O_2_‐induced ROS production in cardiomyocytes

To evaluate the anti‐oxidative effects of ICS II on cardiomyocytes, the cells were injured with H_2_O_2_ for 24 hour at a concentration of 50 nM. The data show that intracellular and mitochondrial ROS levels significantly increased after H_2_O_2_ injury (Figure 3G‐H), but decreased after ICS II treatment. In addition, ICS II treatment reduced the levels of expression of NOX2 and NOX4, and restored HO‐1 (Figure 3I‐L). Moreover, since oxidative stress can lead to cellular apoptosis, we studied the potential anti‐apoptotic effects of ICS II treatment on H_2_O_2_‐injured cardiomyocytes. The data showed that H_2_O_2_ significantly down‐regulated anti‐apoptotic factors Bcl‐2 and up‐regulated pro‐apoptotic factors Bax and cleaved caspase 3. However, ICS II treatment reversed these results (Figure S2B‐D), further indicating that ICS II may play an important role in anti‐oxidative stress and anti‐apoptosis in the context of cardiac hypertrophy.

### 3.5 ICS II suppresses autophagy in TAC‐induced hypertrophy and Ang II–induced hypertrophy

**Figure 4:**
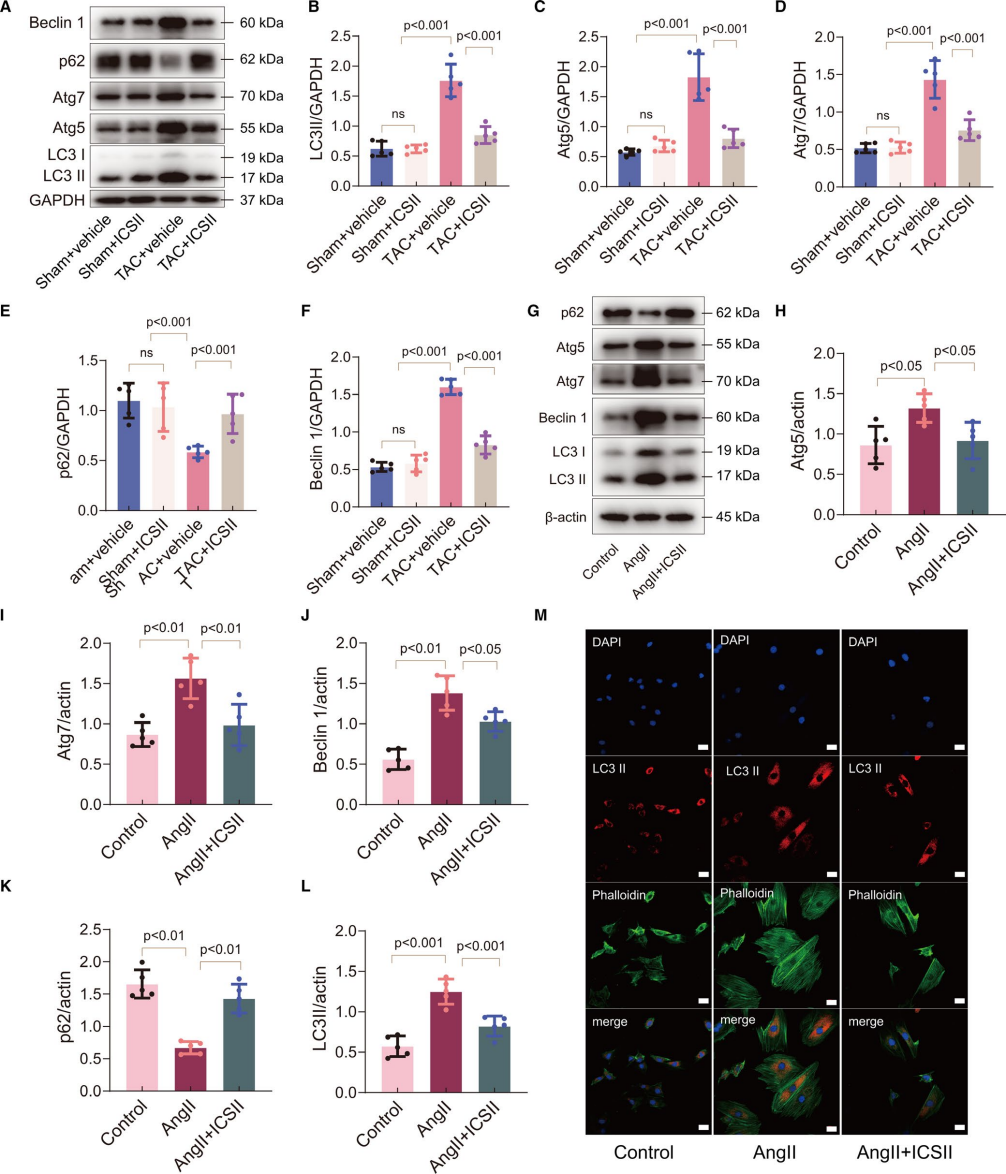
ICS II inhibits autophagy in cardiac hypertrophy. A–F, Western blots for LC3 I and LC3 II, Atg5, Atg7, p62 and Beclin 1, and quantification in *vivo*. n = 5. G–L, Western blots for Atg5, Atg7, Beclin 1, p62, and LC3 I and LC3 II, and quantification in H9C2 cells. n = 5. M, Immunofluorescent micrographs of LC3‐II expression in H9C2 cells. Scale bar = 20μm. n = 3

The possible molecular mechanisms behind the beneficial effects of ICS II were investigated in vivo and in vitro. Autophagic proteins were separated by SDS‐PAGE and quantified. The data indicate that autophagy increased in the mouse myocardium after TAC surgery, and in cells after Ang II was added to the media. In vivo, this increase in autophagy was evidenced by an increase in the LC3‐II/LC3‐I ratio, and in the expression of Beclin 1, Atg5 and Atg7, as well as in the decreased accumulation of p62 (Figure 4A‐L). ICS II treatment reversed the expression of these autophagic markers. In vitro, a similar conclusion was reached by detecting the LC3 II accumulation in cardiomyocytes using immunofluorescence (Figure 4M and Figure S3). These data suggest that ICS II may exert its beneficial effects by inhibiting excess autophagy in the heart after TAC‐ and Ang II–induced cardiac hypertrophy.

### 3.6 Autophagic activation did not block the anti‐hypertrophic effects of ICS II

**Figure 5:**
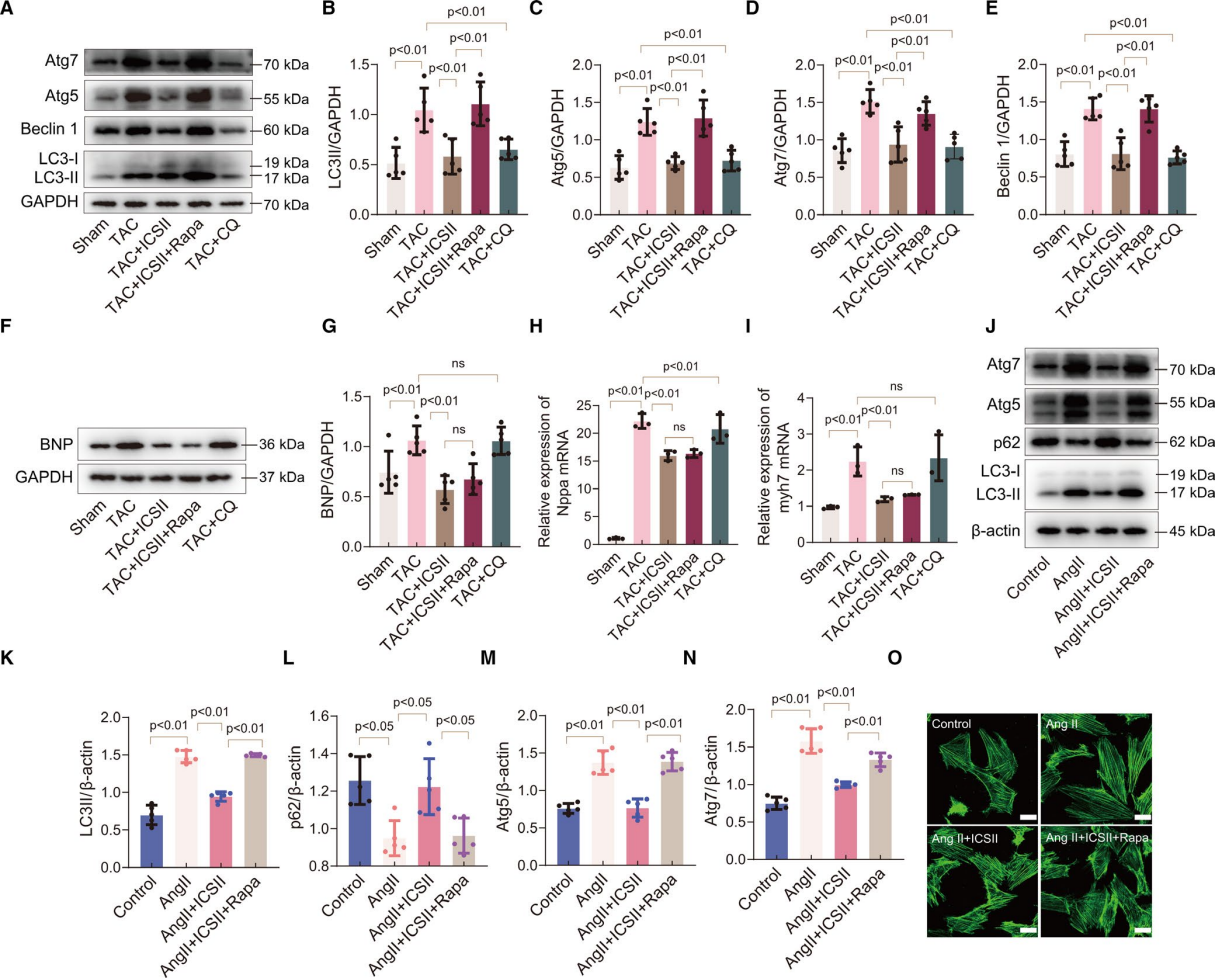
Activating autophagy has no effects on ICS II–mediated anti‐hypertrophy. A–E, Western blots for LC3 II, Atg5, Atg7 and Beclin 1, and quantification in *vivo*. n = 5. F–G, Western blots for BNP, and quantification. n = 5. H, I, Real‐time PCR results for Nppa and Myh7 expression. n = 3. J‐N, Western blots for LC3 II, p62, Atg5 and Atg7, and quantification in *vitro*. n = 5. O, Representative images of phalloidin staining. Scale bar = 20μm. n = 10

Although ICS II inhibits autophagy in vivo and in vitro, we sought to test whether autophagic suppression, by itself, can protect the heart from hypertrophy, and whether ICS II inhibits hypertrophy mainly by inhibiting autophagy. To do this, mice were first treated with rapamycin, an autophagy agonist, after TAC surgery. The data show that TAC‐induced hypertrophic mice that were given rapamycin and ICS II expressed higher levels of autophagic protein markers (LC3 II, Atg5, Atg7 and Beclin 1) than those that were given ICS II but not rapamycin (Figure 5A‐E). The autophagic marker levels in mice that received both ICS II and rapamycin were similar to those in mice that were not treated at all after TAC surgery. By contrast, the expression of BNP protein (Figure 5F‐G), and that of Nppa and Myh7 mRNA (Figure 5H‐I), was not significantly different between the mice in the TAC + ICS II group and those in the TAC + ICS II + rapamycin group. Thus, despite the rapamycin‐mediated rise in autophagy, rapamycin did not block the therapeutic effects of ICS II in the mice. Next, we tested the effects that autophagic suppression would have on cardiac hypertrophy. To do this, we treated the mice with chloroquine, an autophagy inhibitor, after TAC surgery. However, although the expression of autophagic markers decreased, simply inhibiting autophagy did not attenuate cardiac hypertrophy after TAC surgery (Figure 5A‐I). Furthermore, after culturing cardiomyocytes with rapamycin, the elevated autophagic levels did not block the anti‐hypertrophy effects of ICS II (Figure 5J‐O and Figure S4A‐C) and rapamycin has no influence in the protective effects of ICS II when treated with ICS II and rapamycin together (Figure S5). The results indicate that ICS II regulates hypertrophy, and that it decreases autophagy in the myocardium, but it seems that its attenuation of hypertrophy is not dependent on the decreased autophagy.

### 3.7 ICS II increased utilization of ketone bodies and fatty acids in cardiomyocytes

**Figure 6:**
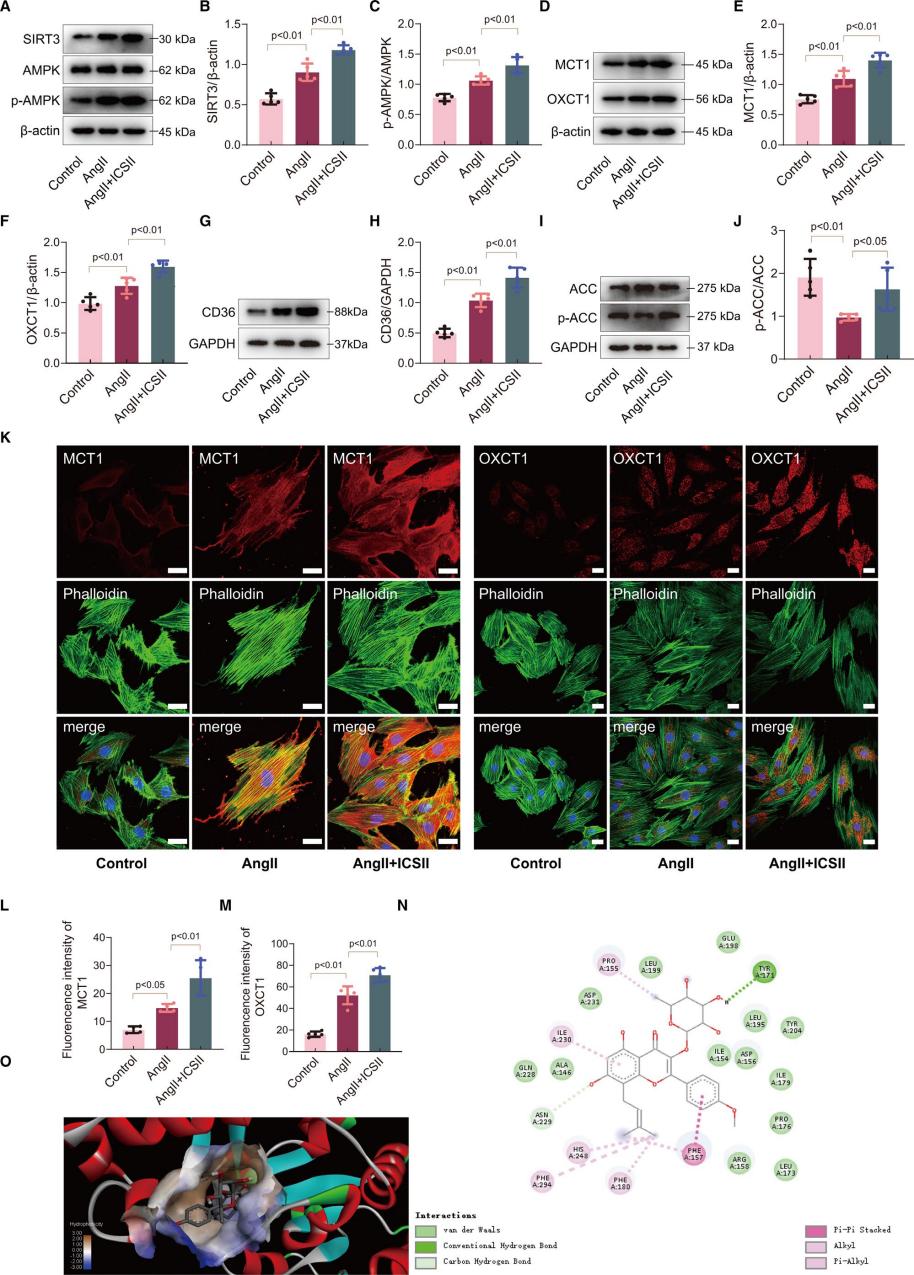
ICS II regulates the energy metabolism of hypertrophic cells. A‐C, Western blots for SIRT3, AMPK and p‐AMPK, and quantification. n = 5. D‐F, Western blots for MCT1 and OXCT1, and quantification. n = 5. G, H, Western blot for CD36 and quantification. n = 5. I, J, Western blot for ACC and p‐ACC and quantification. n = 5. K‐M, Representative immunofluorescent micrographs of MCT1 and OXCT1 expression in vitro, and quantification. Scale bar = 20μm. n = 5

To better understand the mechanisms underlying the therapeutic effects of ICS II on cardiac hypertrophy, we examined SIRT3 and AMPK expression in the cardiomyocytes. ICS II treatment significantly increased the expression of SIRT3 and AMPK proteins in cells with Ang II–induced hypertrophy (Figure 6A‐C). The same is true for their expression of OXCT1, a key enzyme responsible for the metabolism of ketones (by‐products of the breakdown of fatty acids), and MCT1, membrane proteins that act as carriers for ketone bodies. The increase in these key components of ketolysis suggests that ICS II increases ketone body metabolism in response to stress (Figure 6D‐F and K‐M). The data also show that the expression of CD36, a fatty acid transporter, was similarly increased by ICS II treatment (Figure 6G‐H). In addition, the expression of phospho‐acetyl‐CoA carboxylase (P‐ACC), which plays a major role in the oxidation of fatty acids, was significantly increased in cells treated with ICS II (Figure 6I‐J). Taken as a whole, these data suggest that ICS II regulates ketone body utilization and accelerates fatty acid transport and metabolism in hypertrophic hearts through SIRT3‐AMPK activation.

In order to clarify the interaction, we studied the binding affinity of ICS II and SIRT3 using the molecular docking method. The results demonstrated that there is a strong binding affinity between ICS II and SIRT3, with a binding energy of −9.60 kcal/mol. We subsequently explored the possible binding modes and interactions within the amino acid pocket, including TYR171, ASN229, PRO155, PHE157, PHE180, ILE230 and HIS248 (Figure 6N‐O). These results showed that ICS II can directly bind to SIRT3 and effect the way it functions in the context of cardiac hypertrophy.

### 3.8 Genetic down‐regulation of SIRT3 abrogated the effects of ICS II on Ang II–induced hypertrophy and metabolic dysfunction

**Figure 7:**
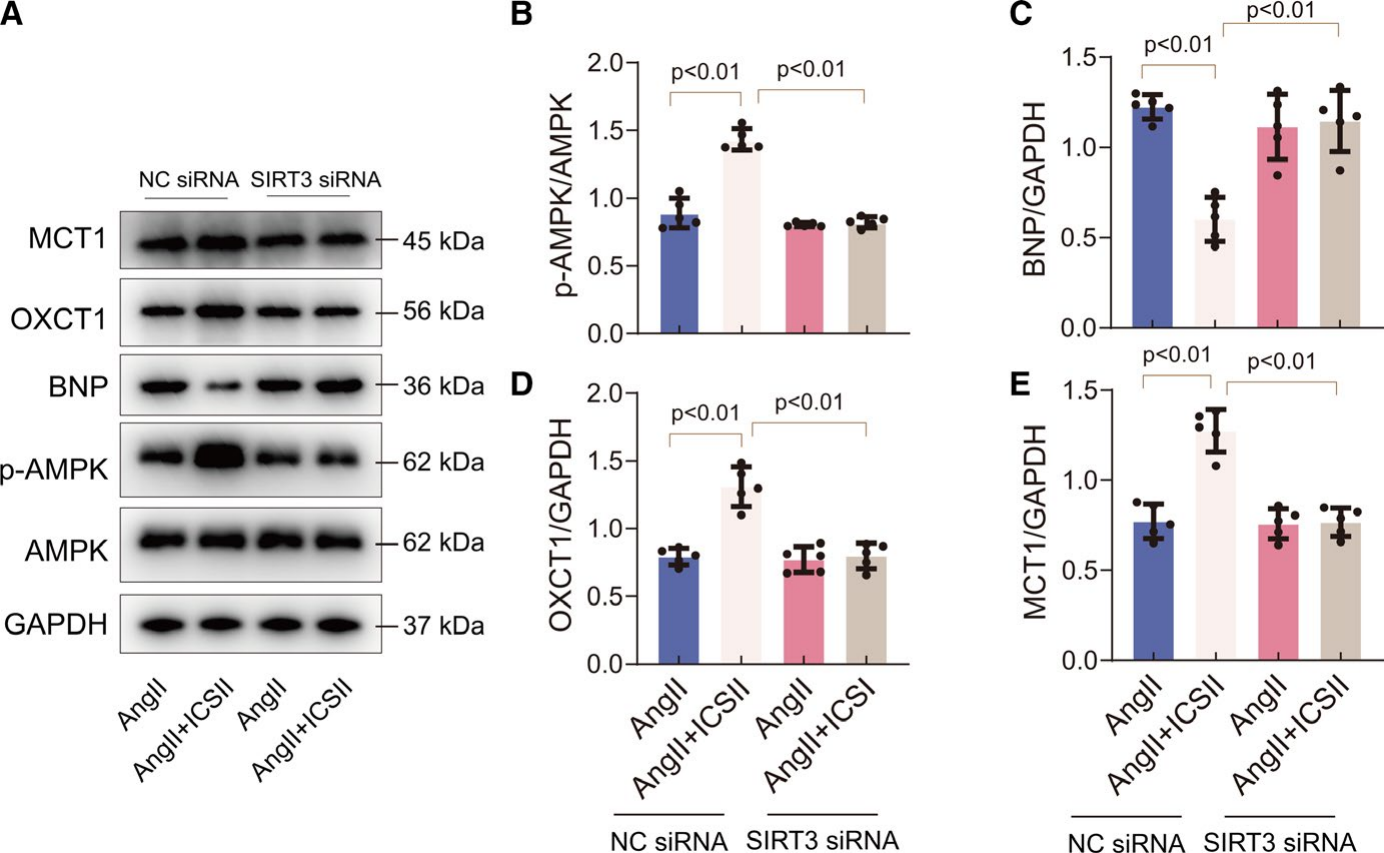
SIRT3 knock‐down reduces the beneficial effects of ICS II on cardiac hypertrophy. A, Western blots for MCT1, OXCT1, BNP, p‐AMPK and AMPK. B‐E, Quantification of p‐AMPK/AMPK, BNP, OXCT1 and MCT1. n = 5

To further investigate whether SIRT3 was involved in the ICS II–mediated suppression of Ang II–induced hypertrophy, we inhibited SIRT3 expression in cardiomyocytes by transfecting them with SIRT3 siRNA. Subsequently, the expression levels of BNP, AMPK/p‐AMPK, OXCT1 and MCT1 proteins in Ang II–induced cardiomyocytes were assessed. As expected, cardiomyocytes transfected with SIRT3 siRNA and cultured with ICS II expressed lower p‐AMPK/AMPK ratios and higher BNP levels than those that were cultured with ICS II but not transfected (NC siRNA) (Figure 7A‐C). Then, the relationship between SIRT3 and ketone body metabolism was explored in Ang II–induced hypertrophic cardiomyocytes treated with ICS II. The data show that SIRT3 knock‐down reduced OXCT1 and MCT1 expression, even after ICS II treatment, compared with the NC siRNA group (Figure 7D‐E). Taken together, these results indicate that the SIRT3‐AMPK pathway plays a mediatory role in the ICS II–mediated protection of Ang II–induced hypertrophic cardiomyocytes.

## 4 DISCUSSION

**Figure 8:**
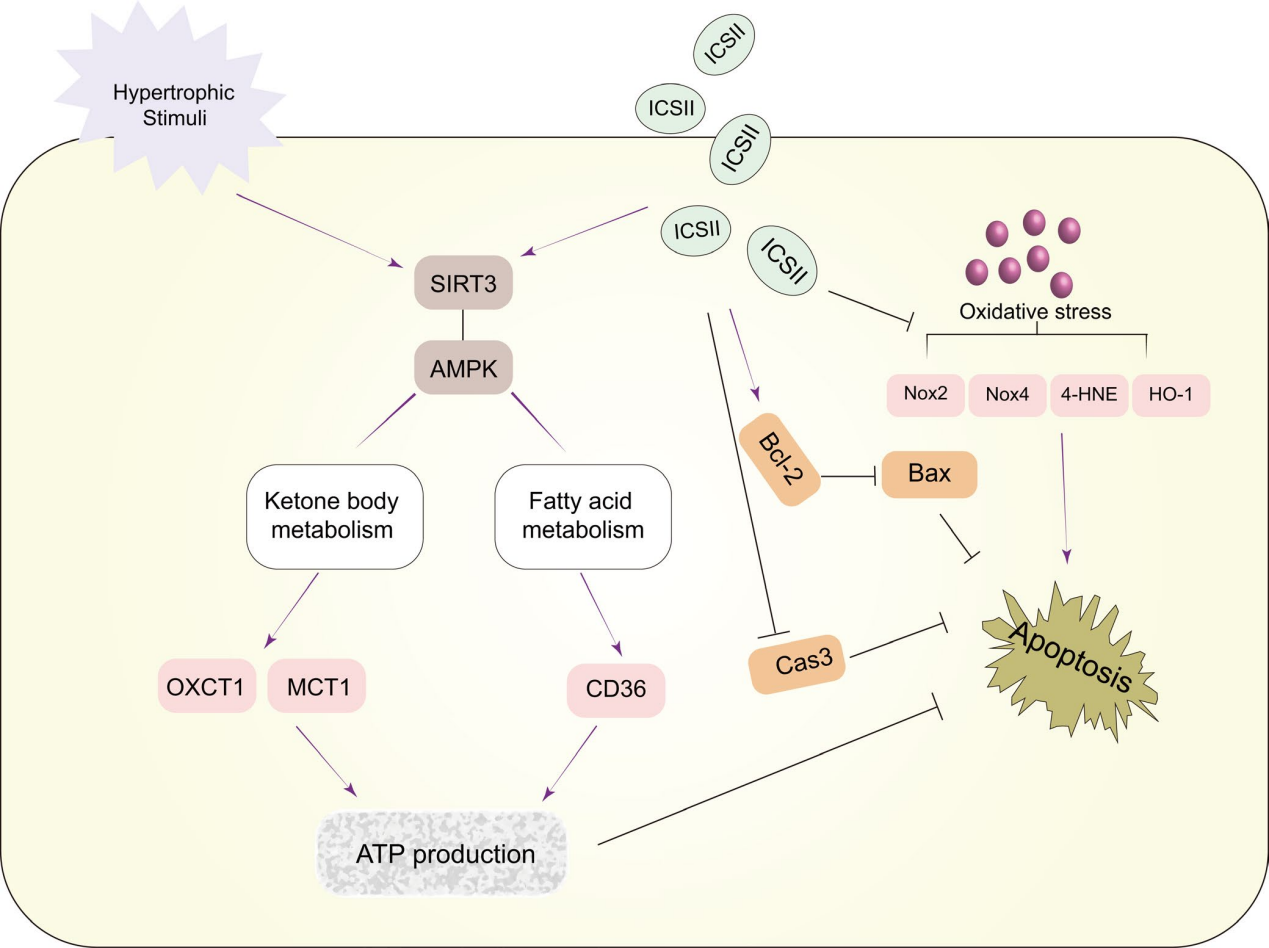
Working model. ICS II inhibits reactive oxygen species production and activates SIRT3 to increase ketone body utilization and preventing cardiac hypertrophy

This study demonstrated the following (Figure 8): (a) ICS II attenuates the pathological markers of cardiac hypertrophy in vivo and in vitro; (b) ICS II inhibits autophagy in TAC and Ang II–induced cardiac hypertrophy; (c) the attenuation of ICS II in cardiac hypertrophy is not due to inhibition of autophagy; (d) ICS II inhibited myocardial metabolic dysfunction by increasing the expression of proteins involved in ketone body and fatty acid metabolism; (e) ICS II regulates cardiac energy metabolism via the SIRT3‐AMPK pathway, as evidenced by the diminished effect of the ICS II treatment on ketone body metabolism when SIRT3 siRNA was used; and (f) ICS II leads to the inhibition of hypertrophic markers.

The medical management of patients with heart failure continues to improve, a trend that is leading to better patient survival rates. However, the morbidity and mortality rates of patients with heart failure remain high.^30^ Previous studies have confirmed that the use of ICS II, a phosphodiesterase‐5 inhibitor, can improve the condition of brains with ischaemic injury.^31^ Other studies show that ICS II improves left ventricular remodelling in spontaneously hypertensive rats.^32^ However, the effects of ICS II on TAC‐induced hypertrophy, and the exact mechanisms of action that lead to potential benefits, still remain unclear. Interestingly, the present study indicates that the treatment of TAC‐induced hypertrophic mice with ICS II has significant cardioprotective benefits. Intermittently administering ICS II for 8 weeks preserved the ejection function of mouse hearts, accompanied by the suppression of cardiac oxidative stress and apoptosis. These findings suggest that ICS II may be a good candidate for clinical translation with the potential to become a viable heart failure therapy. Herein, we look at the mechanisms of action that may be responsible for the therapeutic benefits.

Autophagy is a cellular process that mediates the degradation of misfolded proteins and damaged organelles to maintain cellular homeostasis.^33^ Previous studies indicate that inhibiting excessive cardiac autophagy contributes to the prevention of cardiac hypertrophy.^34^, ^35^, ^36^ Other studies show that autophagy is only slightly elevated for several days after TAC surgery and that enhancing autophagic flux can benefit the heart by helping it adapt to long‐term pressure stimulation.^37^, ^38^, ^39^ Although the role of autophagy is controversial, we found that it was significantly activated in both TAC and Ang II–induced cardiac hypertrophy. In both models of the pathology, autophagic markers LC3, Atg5, Atg7 and Beclin 1 were elevated. They were also accompanied by a decrease in p62 expression, especially in the Ang II–induced hypertrophic model. ICS II treatment down‐regulated the expression levels of LC3, Atg5, Atg7 and Beclin 1. To further clarify whether ICS II exerts its beneficial effects on cardiac hypertrophy through autophagic inhibition, we used rapamycin to induce autophagy in both the TAC‐ and Ang II–induced hypertrophic models. Both the mice (TAC‐induced) and the cardiomyocytes (Ang II–induced) were then treated with ICS II. Interestingly, we did not observe that elevating autophagy blocked the beneficial effects of ICS II on cardiac hypertrophy. We then inhibited autophagy (in mice and cardiomyocytes) with chloroquine to determine whether simply having lower autophagic levels would, by itself, have beneficial effects. However, the data showed that the use of chloroquine did not inhibit the expression of hypertrophic markers BNP, Nppa and Myh7. Collectively, the results indicate that the observed ICS II–mediated prevention of cardiac hypertrophy is not achieved by the direct regulation of autophagy and that simply inhibiting autophagy will not prevent cardiac hypertrophy.

Growing evidence indicates that disorders in the myocardial metabolic process contribute to the development of hypertrophy and heart failure.^40^ To adapt the ATP supply efficiently, the heart shifts its fuel source from fatty acids (FA) to ketone bodies because the latter have a higher oxygen consumption ratio than the former. Clinical research has reported that the treatment of patients with ketone bodies has beneficial haemodynamic effects on their chronic heart failure.^41^ As we expected, ICS II regulated ketone body metabolism by increasing the expression of key enzymes related to ketone body utilization (OXCT1 and MCT1), leading to the attenuation of cardiac hypertrophy. Moreover, ICS II also increased the expression of proteins involved in fatty acid metabolism (p‐ACC and CD36), which suggests that ICS II treatment may increase the availability of metabolic substrates such as fatty acids and ketone bodies in the context of cardiac hypertrophy.

There is mounting evidence that SIRT3 plays a critical role in protecting the heart from cardiac hypertrophy by regulating energy metabolism.^42^, ^43^ SIRT3‐AMPK activation has been shown to regulate glucose uptake in human skeletal muscle cells.^44^ Our data provide evidence that ICS II treatment activates SIRT3 and AMPK in the cells, whereas the genetic down‐regulation of SIRT3 via siRNA leads to a decrease in p‐AMPK/AMPK levels and blunts ketone metabolism. Taken together, our results indicate that ICS II–mediated activation of the SIRT3‐AMPK pathway may be a viable metabolic intervention capable of inhibiting cardiac hypertrophy by increasing ketone body and fatty acid metabolism.

The limitations of the present study are worth noting. First, although the successful application of ICS II in cerebral injury has been well documented, the long‐term safety profile of ICS II use for cardiac hypertrophy, including potential side effects and off‐target effects, needs to be further studied. Second, studies on additional hypertrophic models should be conducted to verify the exact effects of ICS II. Finally, more robust loss‐of‐function and gain‐of‐function studies will need to be conducted to fully assess the relationships between the different players involved in the mechanisms of action.

In conclusion, our study demonstrated that ICS II prevents cardiac hypertrophy induced by TAC and Ang II by regulating metabolic remodelling in the heart through SIRT3‐AMPK activation. Notably, we proved that increasing ketone metabolism may be beneficial for hypertrophic or injured hearts. These findings provide important insights into the molecular mechanisms underlying the cardioprotective effects of ICS II and may help inform the development of novel therapeutic agents that focus on metabolic homeostasis for the treatment of cardiac hypertrophy and heart failure.

## CONFLICT OF INTEREST

The authors declare that they have no conflicts of interest.

## AUTHOR CONTRIBUTIONS

**Dongjian Han:** Conceptualization (equal); Data curation (lead); Methodology (lead); Software (equal); Visualization (lead); Writing‐original draft (lead). **Bo Wang:** Methodology (supporting); Software (supporting); Visualization (supporting). **Xinyue Cui:** Methodology (supporting); Software (supporting); Visualization (supporting). **Weiwei He:** Methodology (supporting); Software (supporting); Visualization (supporting). **Yi Zhang:** Methodology (supporting); Visualization (supporting). **Qingjiao Jiang:** Software (supporting); Visualization (supporting). **Fuhang Wang:** Methodology (supporting); Visualization (supporting). **Zhiyu Liu:** Visualization (supporting). **Deliang Shen:** Conceptualization (equal); Funding acquisition (lead); Project administration (lead); Resources (lead); Writing‐review & editing (lead).

## Supporting information

Fig S1‐5

## Data Availability

The data that support the findings of this study are available from the corresponding author upon reasonable request.
