# Supplementary material for: ICS II protects against cardiac hypertrophy by regulating metabolic remodelling, not by inhibiting autophagy
Source: J Cell Mol Med. 2020 Dec 8;25(2):1074–88. doi: 10.1111/jcmm.16175 (PMC7812268; doi:10.1111/jcmm.16175)
Supplement: Supplementary file 1 — Fig S1‐5 [file JCMM-25-1074-s001.docx]

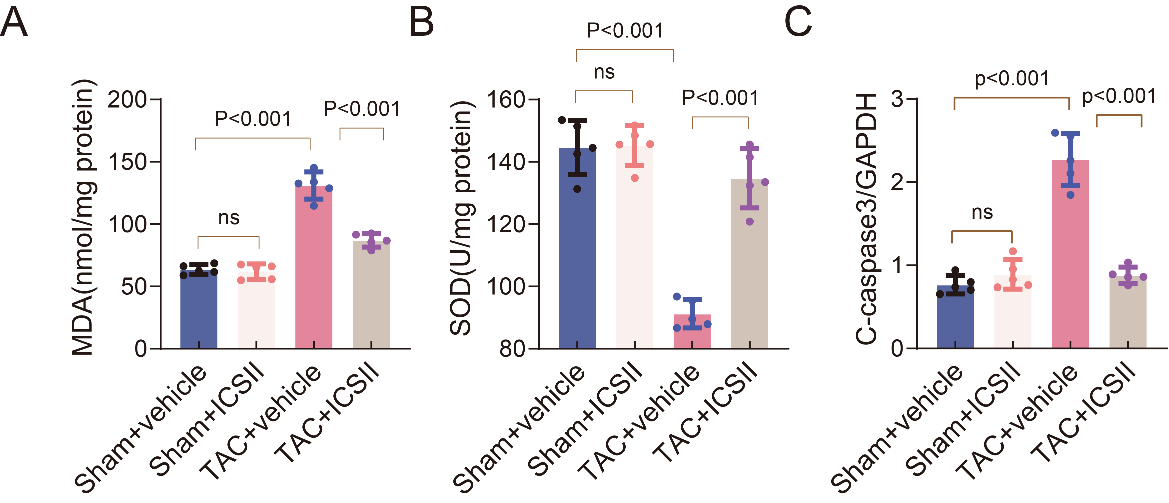


**Figure S1:** Effects of ICS II on myocardial oxidative stress and apoptosis. (A-B) Quantification of SOD and MDA expression in mouse hearts. n = 5. (C) Quantification of cleaved-caspase 3 expression. n = 5. Each column represent the mean ± S.E.M., with n shown above. Differences between means were analysed using one-way ANOVA with Bonferroni post hoc testing . ns, not significant.


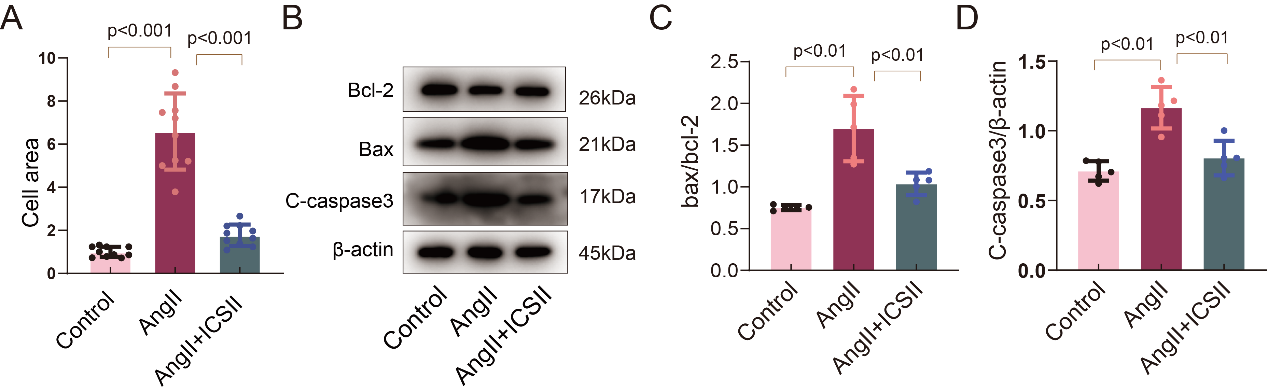


**Figure S2:** ICS II reduced Ang II-induced cardiac hypertrophy and apoptosis induced by Ang II. (A) Quantification of their size (cell area). n = 10. (B-D) Western blots for Bcl-2, Bax, and cleaved-caspase 3, and quantification. n = 5. Each column represent the mean ± S.E.M., with n shown above. Differences between means were analysed using one-way ANOVA with Bonferroni post hoc testing .


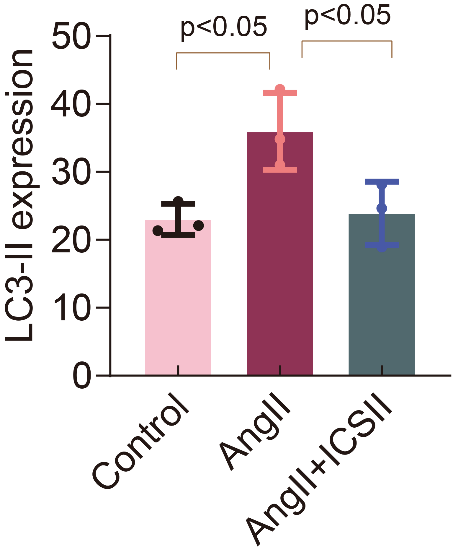


**Figure S3:** Effects of ICS II on regulating autophagy. The quantification of LC3 II expression in H9C2 cells. Each column represent the mean ± S.E.M., with n shown above. Differences between means were analysed using one-way ANOVA with Bonferroni post hoc testing .


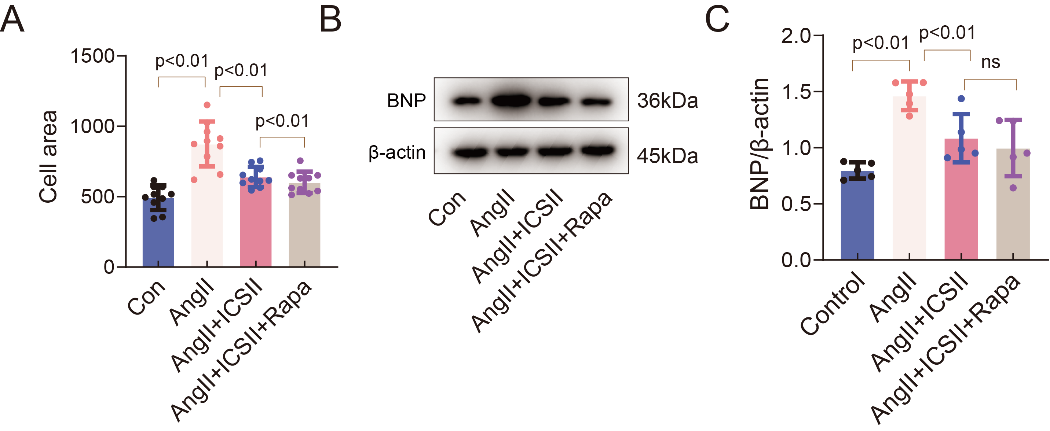


**Figure S4:** Effects of activating autophagy on cell area and BNP expression. (A) Quantification of cell sizes (cell area) in the presence of Rapa. n = 10. (B-C) Western blots for BNP expression, and quantification in vitro. Each column represent the mean ± S.E.M., with n shown above. Differences between means were analysed using one-way ANOVA with Bonferroni post hoc testing .


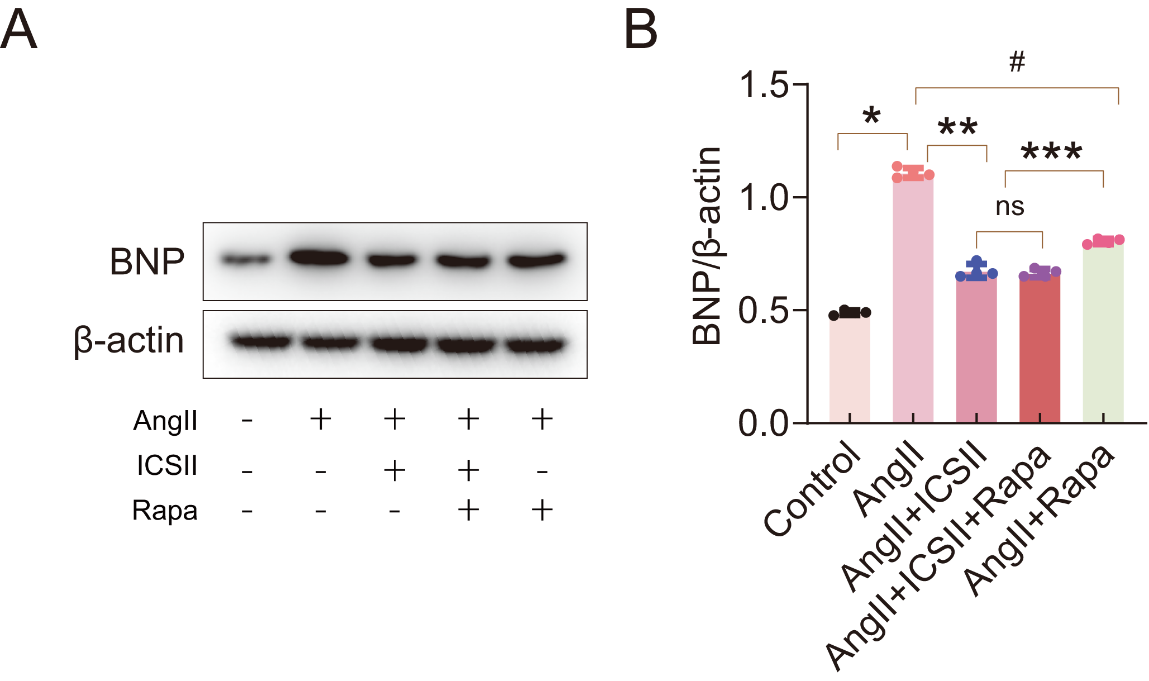


**Figure S5:** Effects of ICS II and Rapamycin on BNP expression in H9C2 cells. (A) Western blot for BNP expression. (B) Quantification of BNP expression. n=4. Each column represent the mean ± S.E.M., with n shown above. Differences between means were analysed using one-way ANOVA with Bonferroni post hoc testing.
